# Apolipoprotein M mediates sphingosine-1-phosphate efflux from erythrocytes

**DOI:** 10.1038/s41598-017-15043-y

**Published:** 2017-11-08

**Authors:** Pernille M. Christensen, Markus H. Bosteen, Stefan Hajny, Lars B. Nielsen, Christina Christoffersen

**Affiliations:** 1 Department of Clinical Biochemistry, Rigshospitalet, Blegdamsvej 9, 2100 Copenhagen, Denmark; 2 Department of Biomedical Sciences, University of Copenhagen, Blegdamsvej 3B, 2200 Copenhagen, Denmark; 3 Department of Clinical Medicine, University of Copenhagen, Blegdamsvej 3B, 2200 Copenhagen, Denmark

## Abstract

Sphingosine-1-phosphate (S1P) is a bioactive lipid implicated in e.g. angiogenesis, lymphocyte trafficking, and endothelial barrier function. Erythrocytes are a main source of plasma S1P together with platelets and endothelial cells. Apolipoprotein M (apoM) in HDL carries 70% of plasma S1P, whereas 30% is carried by albumin. The current aim was to investigate the role of apoM in export of S1P from human erythrocytes. Erythrocytes exported S1P more efficiently to HDL than to albumin, particularly when apoM was present in HDL. In contrast, export of sphingosine to HDL was unaffected by the presence of apoM. The specific ability of apoM to promote export of S1P was independent of apoM being bound in HDL particles. Treatment with MK-571, an inhibitor of the ABCC1 transporter, effectively reduced export of S1P from human erythrocytes to apoM, whereas the export was unaffected by inhibitors of ABCB1 or ATPase. Thus, ABCC1 could be involved in export of S1P from erythrocytes to apoM.

## Introduction

Sphingosine-1-phosphate (S1P) is a small bioactive lipid and a ligand for five G-protein-coupled receptors (S1P_1_-S1P_5_) regulating multiple biological actions. For example, S1P promotes angiogenesis^1,2^, vascular maturation during development, endothelial cell migration, endothelial barrier function^3,4^ and lymphocyte trafficking^5^. S1P is a metabolite from phosphorylation of sphingosine by sphingosine kinase 1 and 2 (Sphk1 and Sphk2)^6,7^. Bone-marrow derived cells, hepatocytes, endothelial cells, platelets, and erythrocytes express Sphk1 or Spkh2 and are potential contributors of plasma S1P^2,5,8,9^. It is of clinical importance to know the detailed regulation of plasma levels of S1P, since this will provide new targets for treatment of diseases affecting for example endothelial barrier function, lymphocyte trafficking, and angiogenesis.

A conditional knockout mouse with lack of both *sphk1* and *sphk2* ^5^ and undetectable plasma S1P regain normal plasma S1P levels after bone marrow transplantation with cells from a WT mouse^5^. Hence, the bone marrow derived cells are able to produce and contribute to plasma S1P. Remarkably, however, transplantation of bone marrow from *sphk1−/−sphk2*+*/−* mice (with 65% reduced plasma S1P) into WT mice did not reduce plasma S1P^9^. This suggests that also non-hematopoietic cells, presumably endothelial cells, can maintain plasma S1P levels. Platelets secrete S1P upon thrombin stimulation^2,10^. Nevertheless, platelet-depleted mice display normal plasma S1P levels^5,9^. In humans, plasma levels of S1P correlate with the hematocrit^11^ and are decreased in patients with anemia^12^, suggesting that erythrocytes contribute to plasma S1P. In accordance with this observation, injection of WT erythrocytes into Sphk-deficient mice restores normal plasma levels of S1P^5^. Altogether, the current evidence suggests that multiple cell types can contribute to plasma S1P, but that erythrocytes likely account for the majority of the S1P production, at least in steady state.

Erythrocytes are unable to release S1P to a plasma- or serum-free medium. Hence, an acceptor needs to be present in order to promote S1P export^13^. In plasma, S1P is bound to albumin (~30%) or HDL particles (~70%)^14,15^, and both albumin and HDL can act as acceptors of S1P exported from erythrocytes^12,16^. The export of S1P from erythrocytes to albumin can be inhibited by glyburide (an inhibitor of the ATP-binding cassette transporter A1 (ABCA1))^17^, vanadate (a phosphate analogue and inhibitor of several ATPases)^17^, and 1,4-dithioerythritol (an inhibitor of scramblase activity)^12^. In other cell types SPNS2^18^ and ABCC1^19^ can mediate export of S1P, however they have not been established as transporters in erythrocytes. It is unknown whether S1P export to HDL involves ABC transporters and whether S1P is accepted by the lipid moiety of the HDL particles or specific HDL apolipoproteins.

HDL contains more than 40 different proteins. So far it has been shown that apoAI^16^, apoCI, or apoCII^12^ cannot promote S1P export from erythrocytes. Recently, apoM was identified as the physiological carrier of S1P in plasma^20^. ApoM is a lipocalin and has a characteristic beta-barrel structure enclosing a hydrophobic binding pocket, which accommodates S1P^20^. ApoM is mainly associated with HDL particles (~5% of HDL particles contain an apoM molecule^21^). Thus, apoM-free HDL in humans does not contain any detectable S1P, and lack of apoM/S1P compromises the pulmonary endothelial barrier in mice, suggesting a unique biological role for apoM/S1P-containing HDL^20^. ApoM is expressed in the liver and kidney^22^, and apoM seems to play a significant role in S1P export from the liver^23^. However, erythrocytes are probably the main contributor of plasma S1P. It is unknown whether and how erythrocyte-produced S1P is transferred to apoM.

The purpose of this study was to explore to what extent apoM can serve as an acceptor of S1P from erythrocytes and to identify potential transporters involved in erythrocyte export of S1P to HDL.

## Results

### Isolation of HDL with and without apoM

In order to investigate the effects of apoM in human HDL on export of S1P from human erythrocytes, we developed an affinity column where monoclonal antibodies specific for human apoM were generated and coupled to a resin column. By passing purified human HDL over the column, we could isolate apoM-free human HDL-apoM from the flow-through and subsequently elute apoM-containing human HDL + apoM from the column. Of note, the protein composition of HDL-apoM and HDL + apoM with regards to apoAI and apoAII, as analyzed with silverstained SDS-PAGE, was similar and human apoM was exclusively detected (by western blotting and ELISA) in HDL + apoM (see Supplemental Fig. S1). The molar ratio of S1P to apoM was found to be 1:5.5 for HDL + apoM. S1P was not detectable with a sensitive HPLC method in HDL-apoM.

HDL with and without apoM was isolated from human apoM transgenic (apoM-Tg^H^) mice with 10-fold increase in human plasma apoM and apoM^−/−^ mice without apoM^24^, respectively. The HDL particles contained similar levels of several apolipoproteins including apoAI and apoAII, as described previously^25^. Previous analysis showed that the molar ratio of S1P to apoM is ~1:6 for HDL from apoM-Tg^H^ mice and HDL from apoM^−/−^ mice contains no detectable S1P^20^.

### Export of S1P from erythrocytes to albumin and HDL

**Figure 1:**
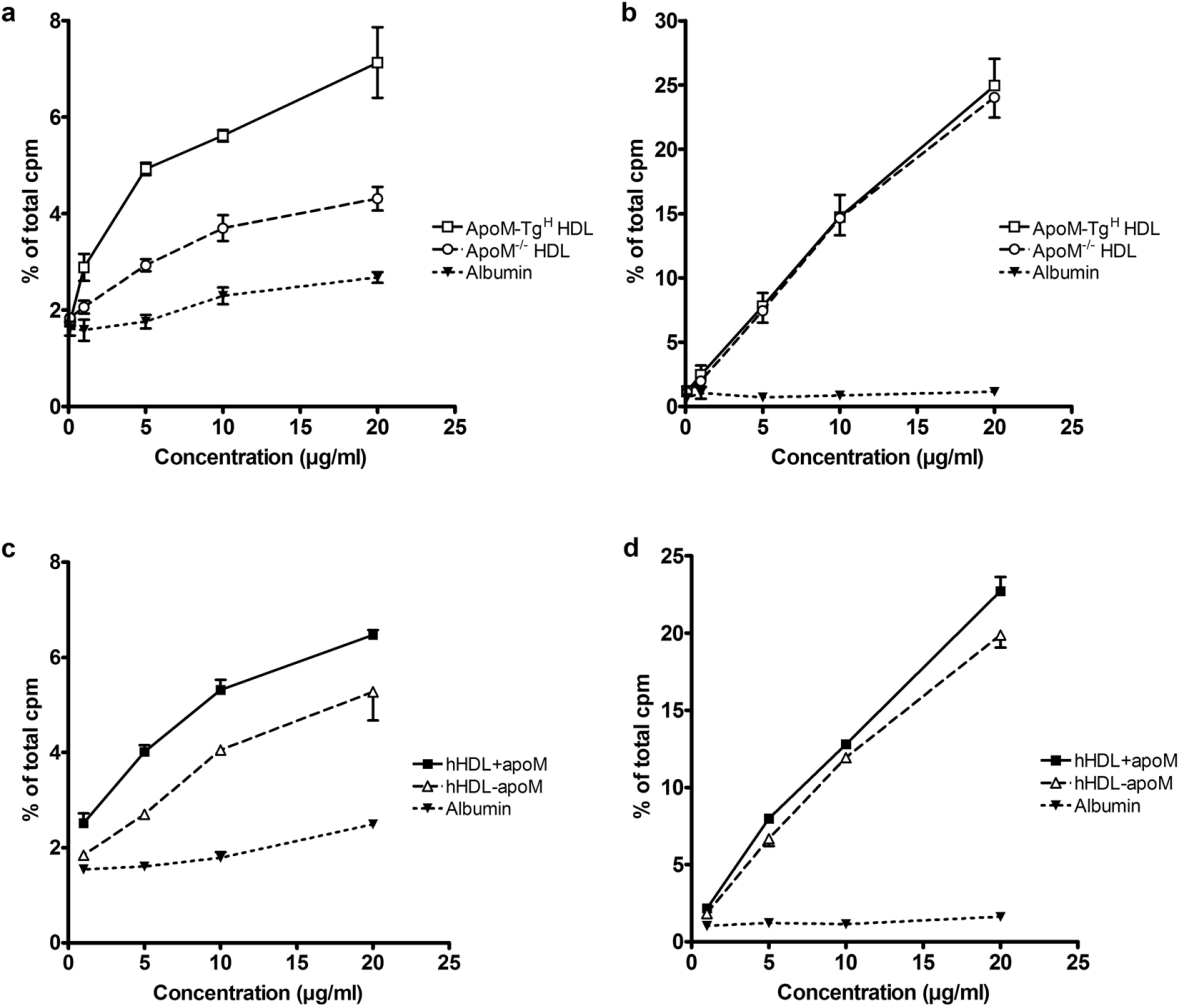
ApoM-containing HDL increases export of S1P but not sphingosine from erythrocytes. Export of S1P (**a** and **c**) and export of sphingosine (**b** and **d**) from erythrocytes incubated 40 min with HDL from apoM transgenic mice (apoM-Tg^H^), HDL from apoM^−/−^ mice, and albumin (**a** and **b**) or human HDL + apoM, human HDL-apoM, and albumin (**c** and **d**). Results are expressed as % of the total cpm found in supernatants and pellets combined and plotted as mean ± SEM.

We first compared murine HDL with or without apoM and albumin as acceptors of S1P produced and secreted from human erythrocytes. ^3^H-sphingosine-loaded erythrocytes were incubated with albumin, HDL from apoM-Tg^H^ mice or HDL from apoM^−/−^ mice. At all protein concentrations tested (0.1–20 μg/ml total protein) more ^3^H-S1P was exported to HDL (1.75 ± 0.03% to 7.13 ± 0.73% for apoM-Tg^H^ HDL and 1.84 ± 0.09% to 4.31 ± 0.25% for apoM^−/−^ HDL) than to albumin (1.62 ± 0.14% to 2.68 ± 0.11%) (Fig. 1a). The presence of human apoM in mouse HDL conferred a larger ^3^H-S1P export compared to HDL without apoM (7.13 ± 0.73% vs. 4.31 ± 0.25% at 20 μg/ml, p = 0.02) (Fig. 1a). The presence of apoM in human HDL also increased the ability of HDL to mediate export of ^3^H-S1P from erythrocytes (5.32 ± 0.22% vs. 4.05 ± 0.02%, at 10 µg/ml, p = 0.028) (Fig. 1c). Of note, even when the albumin concentration was increased to 500 µg/ml, HDL + apoM and HDL-apoM were still superior as mediators of ^3^H-S1P export (Supplemental Fig. S2). Hence, a 10 times higher albumin concentration induces considerable less ^3^H-S1P release as when compared to HDL.

**Figure 2:**
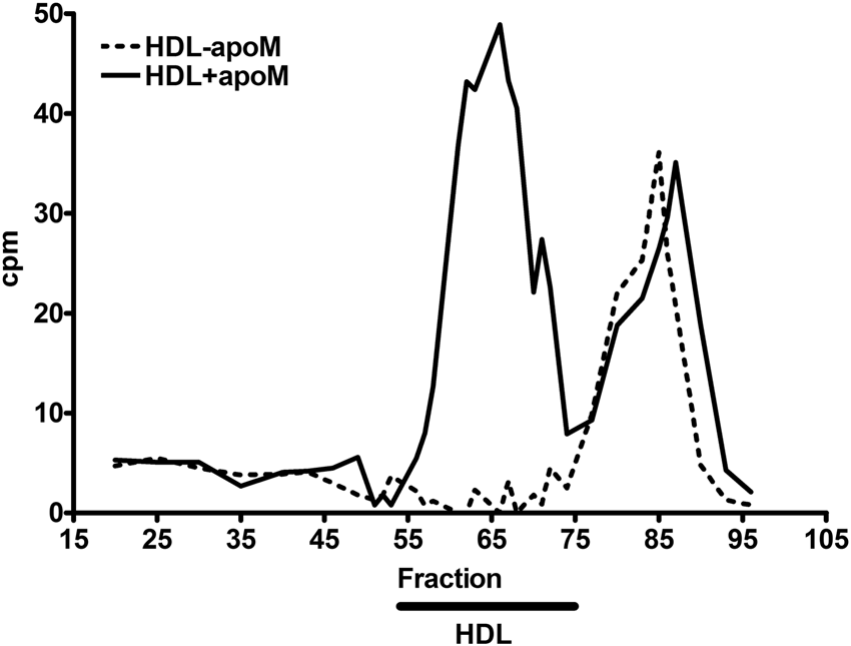
S1P is only bound to HDL particles containing apoM. Measurement of ^3^H-S1P in gel filtration fractions from export assays with human HDL + apoM (^**_______**^) or HDL-apoM (- - - - -) as acceptors (10 µg/ml). Radioactivity in the selected fractions is shown as cpm after correction for background activity. The results shown are representative of two separate experiments. The black bar indicates fractions where HDL particles are eluted.

**Figure 3:**
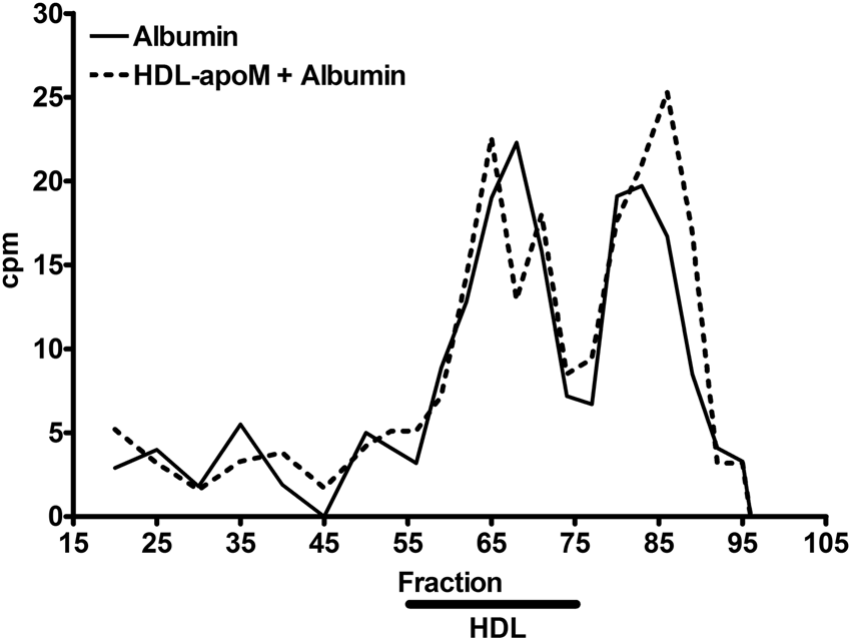
S1P exported to HDL-apoM can associate with albumin. Measurement of ^3^H-S1P in gel filtration fractions from export assays with 500 µg/ml albumin (^______^) or 10 µg/ml human HDL-apoM (- - - - -) as acceptors. After export, supernatants from assays with HDL-apoM were mixed with 500 µg/ml albumin and allowed to incubate for 10 minutes at 37 °C before subjected to gel filtration. Radioactivity in the selected fractions is shown as cpm after correction for background activity. The black bar indicates fractions where HDL particles are eluted.

In plasma, S1P is only found in HDL particles containing apoM^20^ and thus it was surprising that human and murine HDL without apoM could facilitate export of S1P from human erythrocytes. To assess if the effluxed ^3^H-S1P was bound to HDL without apoM, supernatants from ^3^H-sphingosine-loaded human erythrocytes incubated with human HDL with apoM or human HDL without apoM were subjected to gel filtration. As expected, when ^3^H-sphingosine-loaded erythrocytes had been incubated with HDL with apoM, ^3^H-S1P was mainly detected in the apoM-containing HDL particles (a smaller fraction was recovered as unbound ^3^H-S1P) (Fig. 2 and Supplemental Fig. S3). Remarkably, however, when ^3^H-sphingosine-loaded erythrocytes had been incubated with HDL without apoM the ^3^H-S1P was almost exclusively detected in fractions corresponding to unbound S1P or in fractions with low concentration of apoA-I and albumin (Fig. 2 and Supplemental Fig. S3). Interestingly, if albumin was added afterwards to the supernatant from export assays using HDL-apoM as acceptor, ^3^H-S1P was recovered in fractions similar to experiments using albumin as acceptor (Fig. 3).

**Figure 4:**
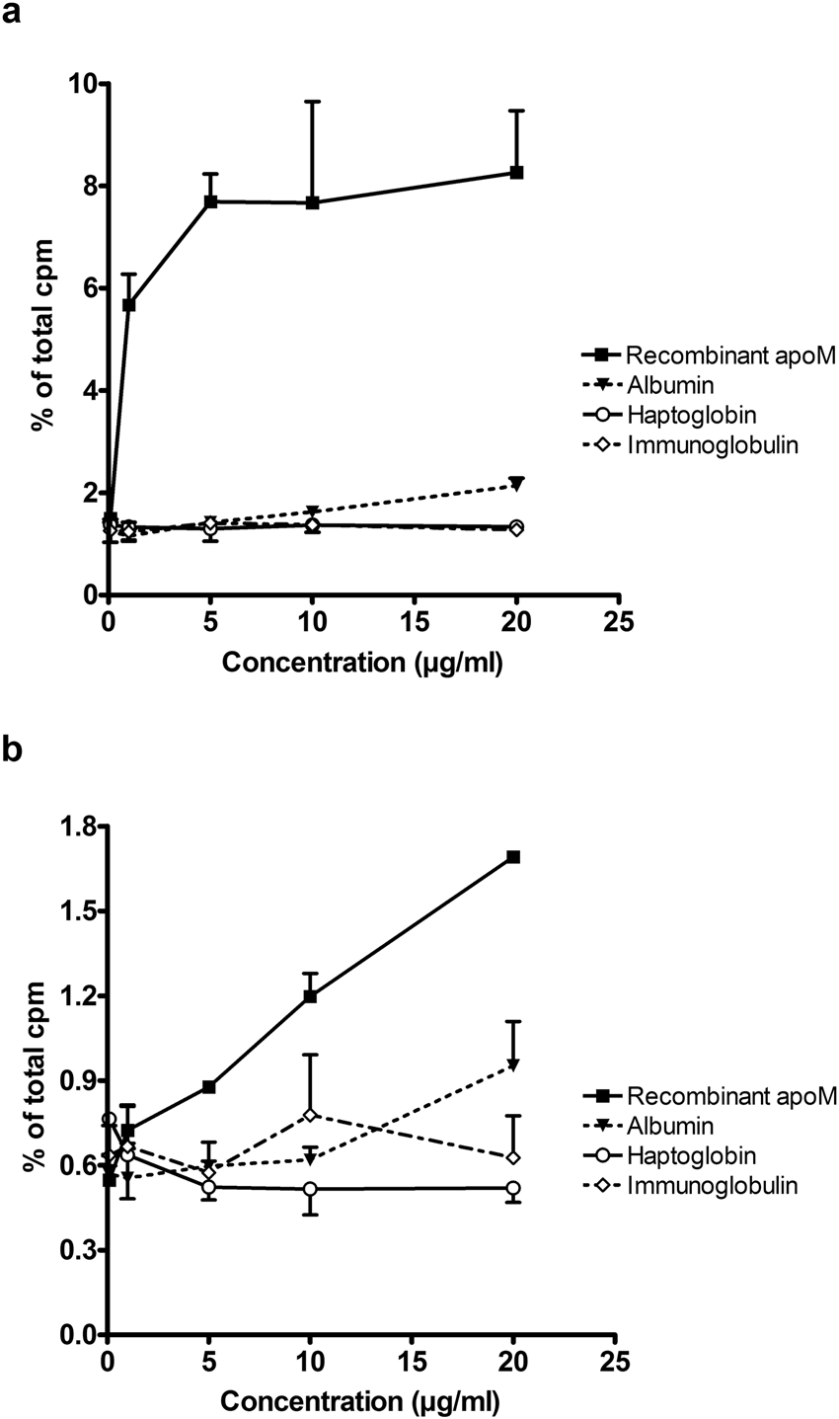
Export of S1P from erythrocytes requires presence of physiological carrier proteins. Export of S1P (**a**) and export of sphingosine (**b**) from erythrocytes incubated 40 min with recombinant apoM protein, albumin, haptoglobin, or immunoglobulins. Results are expressed as % of the total cpm in supernatants and pellets combined and plotted as mean ± SEM.

**Figure 5:**
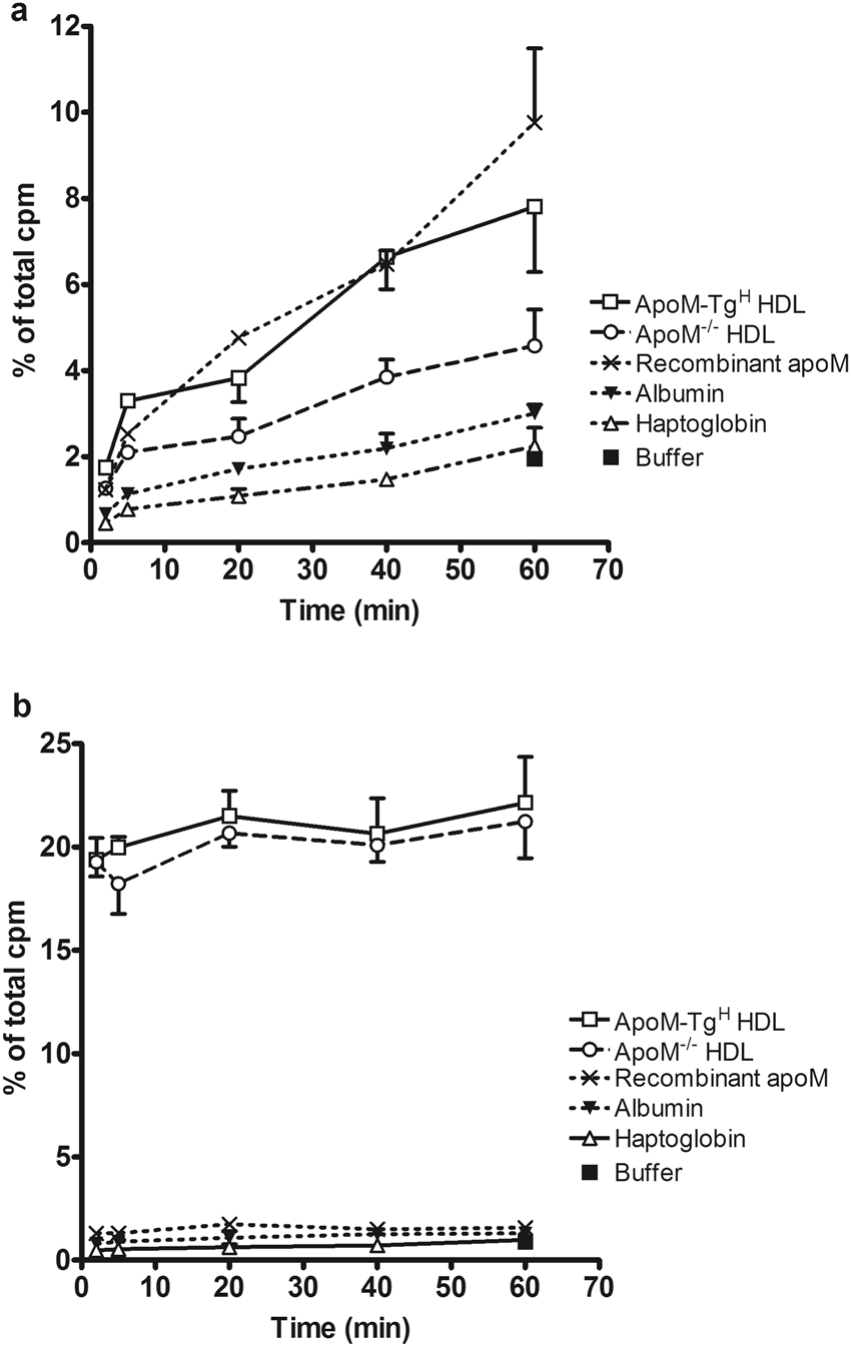
ApoM facilitates increased S1P export over time. Export of S1P (**a**) and sphingosine (**b**) from erythrocytes incubated with HDL from apoM transgenic mice (ApoM-Tg^H^), HDL from apoM^−/−^ mice, recombinant apoM protein, albumin, haptoglobin, or assay buffer without any protein. Total protein concentration 20 µg/ml and samples were incubated 2 to 60 minutes. Results are expressed as % of the total cpm in supernatants and pellets combined and plotted as mean ± SEM.

Since apoM-containing HDL from both mice and humans facilitated increased export of S1P from erythrocytes as compared to apoM-free HDL, it was of interest to investigate whether apoM needed to be in complex with HDL in order to promote S1P export. Hence, we assessed whether a truncated recombinant apoM protein (167 amino acids), lacking its HDL binding signal peptide (21 amino acids) but retaining its ability to bind S1P, could mediate S1P export from erythrocytes. Thus, ^3^H-sphingosine-loaded erythrocytes were incubated with the recombinant apoM protein, albumin, haptoglobin, or immunoglobulins. At all concentrations tested (0.1–20 μg/ml) recombinant apoM protein was superior in promoting export of ^3^H-S1P compared to albumin (8.26 ± 1.21% vs. 2.14 ± 0.15%, at 20 µg/ml, p = 0.037) (Fig. 4a). Furthermore, in direct comparison with apoM-containing HDL from Tg^H^ mice, recombinant apoM protein promoted similar time-dependent (2–60 minutes) export of ^3^H-S1P from human erythrocytes (Fig. 5a). In a separate experiment, with prolonged incubation for up to 6 hours, recombinant apoM even lead to the highest amount of ^3^H-S1P exported after 6 hours (28.80%) followed by apoM-Tg^H^ HDL (17.43%), apoM^α^ HDL (11.50%), albumin (6.81%) and haptoglobin (6.57%) (see Supplemental Fig. S4).

### Erythrocyte export of sphingosine

To determine whether apoM promoted a general export of sphingolipids or whether the export was specific for S1P, we measured the export of ^3^H-sphingosine from human erythrocytes. The erythrocytes exported considerably higher amounts of ^3^H-sphingosine when incubated with HDL compared to incubation with albumin (24.95 ± 2.08% vs. 1.15 ± 0.10% at 20 μg/ml, p = 0.0003), and the export of ^3^H-sphingosine to HDL was not affected by apoM (Fig. 1b,d). Recombinant apoM protein was slightly more effective in promoting export of ^3^H-sphingosine (1.69 ± 0.02% at 20 μg/ml) compared to albumin (0.95 ± 0.16% at 20 μg/ml), haptoglobin (0.52 ± 0.05% at 20 μg/ml) or immunoglobulins (0.63 ± 0.15% at 20 μg/ml) (Fig. 4b). However, when compared to HDL particles, all of the tested proteins were markedly less efficient in promoting ^3^H-sphingosine export (compare Figs 1b and 5b).

### S1P export from erythrocytes is inhibited by MK-571, an ABCC1 inhibitor

**Figure 6:**
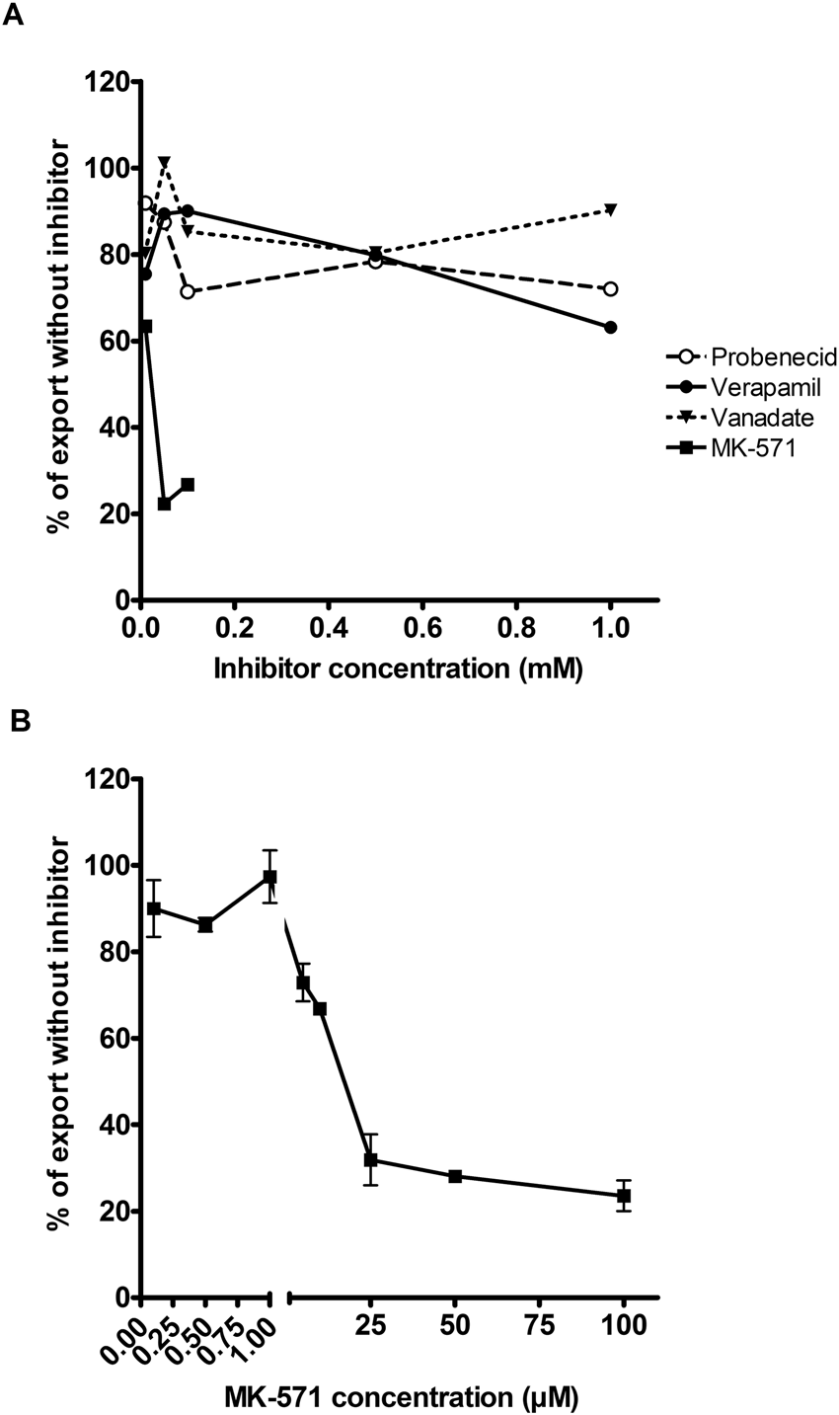
S1P export from erythrocytes is inhibited by an ABCC1 inhibitor MK-571. Export of S1P from erythrocytes incubated with recombinant apoM protein (20 µg/ml) in (**A**) the initial screening of various concentrations of Probenecid, Verapamil, Vanadate, and MK-571 and (**B**) screening of additional concentrations of MK-571. In (**A**) concentrations of MK-571 above 0.1 mM led to hemolysis and were excluded. Results are expressed as % of the export seen from erythrocytes incubated without inhibitor. In (**B**) the results are plotted as mean ± SEM.

To investigate transporter pathways involved in S1P export from erythrocytes to apoM, several inhibitors of ABC transporters were screened for their effect on ^3^H-S1P export to recombinant apoM protein. Probenecid (inhibitor of several ABCC transporters), Verapamil (inhibitor of ABCB1), and Vanadate (ATPase inhibitor) all had limited inhibitory effect on ^3^H-S1P export (maximum inhibition 29%, 37%, and 20%, respectively) at the concentrations tested (0.01–1 mM) (Fig. 6a). In contrast, the ABCC1 inhibitor MK-571 effectively reduced ^3^H-S1P export to the medium by 88% (50 μM) in the initial screening. Further experiments showed that MK-571 reduced the export of ^3^H-S1P from erythrocytes in a concentration-dependent manner (0.01–100 μM) (Fig. 6b).

### S1P export is not altered in erythrocytes from Abcc1-deficient mice

**Figure 7:**
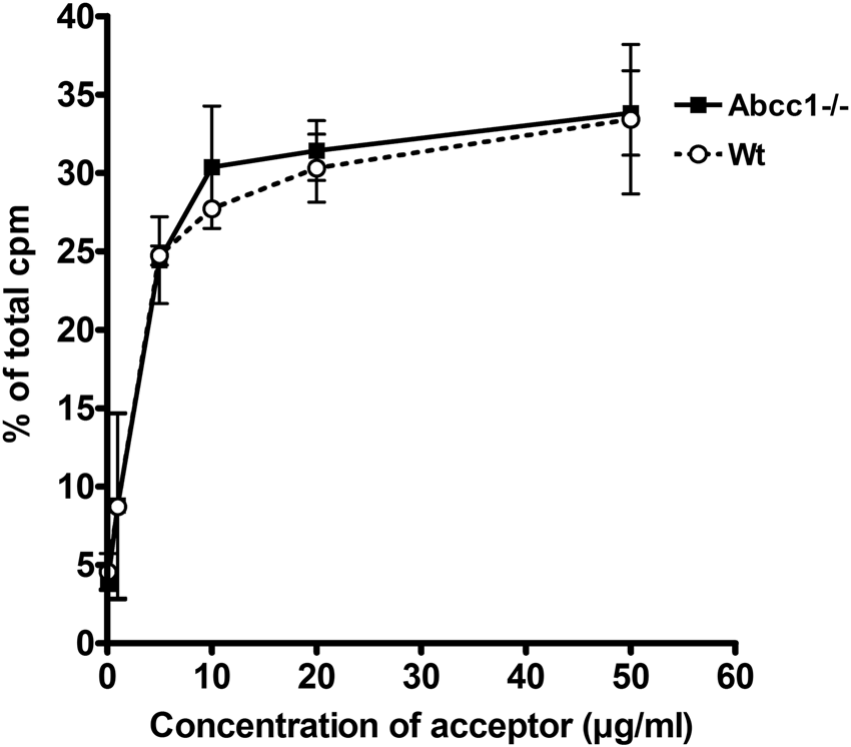
S1P export from ABCC1-deficient murine erythrocytes is unaltered. Export of S1P from erythrocytes isolated from Abcc1−/− or Wt mice after incubation for 40 min with recombinant apoM (0–50 µg/ml). Results are expressed as % of the total cpm measured in supernatants and pellets combined. The experiment was repeated twice and data are plotted as mean ± SEM.

To further investigate the role of ABCC1 in export of S1P from erythrocytes, we isolated erythrocytes from Abcc1-deficient mice and their wildtype controls. When the Abcc1-deficient erythrocytes were used in the S1P export assay, we observed no difference in S1P export to recombinant apoM protein compared to erythrocytes from wildtype controls (Fig. 7).

## Discussion

The present data suggest that HDL promotes export of S1P from human erythrocytes compared to albumin and that this is further enhanced when apoM is present in the HDL particles. Furthermore, we find that apoM can effectively promote export of S1P independently of its association with HDL, i.e. as free recombinant protein, and that the ABCC1 transporter could be involved in export of S1P from human erythrocytes to apoM. Finally, we show that export of sphingosine from erythrocytes is unaffected by the presence of apoM in HDL.

We have identified that apoM-containing HDL increases the export of S1P from human erythrocytes compared to apoM-free HDL and albumin. These observations are supported by a recent study by Sutter *et al*.^16^, despite the use of two different experimental setups. They showed that incubation of erythrocytes with HDL from apoM transgenic mice increased accumulation of S1P in the medium compared to incubation with HDL from WT or apoM^−/−^ mice or incubation with albumin. Sutter *et al*.^16^ incubated erythrocytes with the various acceptors and measured total S1P content in the medium whereas the present study used erythrocytes preloaded with ^3^H-labelled sphingosine and subsequent measured ^3^H-sphingosine and ^3^H-S1P in the medium and cells. With this experimental setup we found that HDL with apoM isolated from both human and mouse plasma increased the export of ^3^H-S1P from human erythrocytes compared to HDL without apoM. The difference between HDL with apoM and HDL without apoM seemed to be more pronounced for murine HDL than human HDL. This could be not explained by the S1P to apoM ratio, which was similar for murine Tg^H^ HDL and human HDL + apoM.

In plasma, HDL-S1P is only detectable in the apoM containing fraction^20^. Therefore, it was somewhat surprising that even HDL without apoM mediated ^3^H-S1P export from human erythrocytes, albeit less effective than HDL with apoM. Notably, this observation was not due to cross contamination of ^3^H-sphingosine since the currently employed separation of ^3^H-S1P and ^3^H-sphingosine was almost complete with 99% recovery of ^3^H-S1P, as described in the methods section. Moreover, the notion that HDL without apoM is capable of inducing export of S1P from erythrocytes is in agreement with the S1P mass measurement by Sutter *et al*.^16^. The present study suggests that even though S1P is efficiently exported in presence of HDL without apoM, it is not bound to the HDL particles when apoM is not present. This is in line with *in vivo* observations, where S1P is undetectable in apoM-free HDL^20^. It is possible that S1P associates weakly with apoM-free HDL, probably by binding to the lipid moiety, during the export process and subsequently is transferred to either apoM-containing HDL or albumin *in vivo*. Our data support that S1P exported to HDL without apoM can be transported to albumin if present in sufficient amounts. Furthermore, since HDL with or without apoM both are able to induce S1P export from erythrocytes to a significantly larger extend than albumin, other apolipoproteins than apoM could serve as co-factors driving the export process. The identity of such co-factor still remains to be determined.

The biological roles of S1P are multiple. S1P regulates angiogenesis, migration of endothelial cells, vascular development, and enhancement of the endothelial barrier among others. Hence, to understand the metabolism of S1P it is important to including possible regulated transport mechanisms. Therefore, efforts have been made to identify the transporter(s) responsible for exporting S1P from erythrocytes. Transport of S1P in inside-out vesicles from rat erythrocytes is ATP-dependent and inhibited by Glyburide, an ABCA1 inhibitor^17^. However, ABCA1 expression was undetectable in the inside-out vesicles and thus excluded as the responsible S1P transporter^17^. Further, Vanadate, a phosphate analogue that inhibits several ATPases, also inhibited release of S1P^17^. These observations were however challenged by a second study which showed no decrease in S1P export from human erythrocytes after treatment with Vanadate^12^. Instead a scramblase inhibitor effectively reduced the export of S1P from erythrocytes^12^. We propose that the ABCC1 transporter is involved in the export of S1P, since treatment with MK-571 (an ABCC1 inhibitor) significantly inhibits release of S1P from human erythrocytes to recombinant apoM protein. The ABCC1 transporter could be a candidate, supported by the observation that ABCC1 also is responsible for export of S1P in mast cells^19^. However, MK-571 had no effect on S1P release from intact rat erythrocytes nor from inside-out vesicles^17^. Reasons for the deviations could be that Kobayashi *et al*. used rat erythrocytes and albumin, whereas we have used human erythrocytes and recombinant apoM protein as an S1P acceptor. In our hands, albumin as acceptor release substantially lower amounts of S1P from erythrocytes compared to recombinant apoM protein or HDL. Thus, the effects of MK-571 could be blurred when using albumin as an acceptor since secretion of S1P to albumin is minimal already without use of inhibitor. Also, it cannot be excluded that different export mechanisms exist for export to albumin and apoM or for export in rat and human erythrocytes. It is already known that there is functional diversity between S1P-albumin and S1P-apoM-containing HDL. For example, to initiate tight junction formation via S1P_1_ the S1P-apoM complex is needed, whereas the S1P-albumin complex *in vitro* and *in vivo* is not able to induce similar levels of S1P_1_ mediated tight junctions causing increased vascular permeability^20^.

In our initial screening Probenecid only led to limited inhibition of S1P export. Probenecid is an inhibitor targeting multiple ABCC transporters; hence an inhibitory effect comparable to the effect seen with MK-571 would be expected. It is possible that the concentrations used of Probenecid were insufficient to get maximal inhibition, that the incubation time was too short or that the affinity of Probenecid to ABCC1 was lower than to the other ABCC transporters. Additionally, we analyzed ABCC1 inhibition with Cediranib, a VEGF receptor tyrosine kinase inhibitor, described in one study to have inhibitory effects on ABCC1 in human carcinoma cell lines^26^. However, the results showed no inhibition of S1P export from human erythrocytes (data not shown), indicating thatABCC1 is not responsible for S1P transport in human erythrocytes or that Cediranib is not effective as ABCC1 inhibitor in our experimental setup.

Surprisingly, erythrocytes from ABCC1-deficient mice retained the ability to export S1P in the presence of apoM. This suggests that the ABCC1 transporter is not essential for export of S1P from murine erythrocytes. In human erythrocytes, our results with MK-571 indicate that ABCC1 may play an essential role in S1P release. MK-571 is, however, also a leukotriene D_4_ antagonist^27^ and thus could potentially interfere with the general lipid signaling in the erythrocytes, thereby affecting the export of S1P. To further elucidate the role of ABCC1 in export of S1P, development of specific ABCC1 inhibitors are needed. Taken together, more experiments are needed to definitively determine both the significance of human ABCC1 in S1P export, and the species specific role of of apoM.

Clinically, a further development of MK-571, Montelukast, is used in prophylactic treatment of patients with asthma. Based on our observations that MK-571 inhibits the secretion of S1P from human erythrocytes, it could be interesting to determine if plasma levels of S1P in these patients are altered. Further, even though we only observed a modest effect of Verapamil and Probenecid on S1P export, these compounds are used clinically in treatment of arrhythmias and gout, respectively. Thus, determination of plasma S1P levels in these patient groups could also be of interest. To our knowledge, this has yet to be done. S1P is a potent bioactive lipid, involved in angiogenesis, endothelial barrier function, and lymphocyte migration among others. Hence, to be able to target treatment for control of plasma S1P levels will be of great clinical value.

In conclusion we show that HDL promotes increased export of S1P from human erythrocytes and that this is further enhanced by the presence of apoM. Also, apoM induces S1P export from erythrocytes independently of binding to HDL particles. Our observations are supported by previous findings from Sutter *et al*.^16^ despite two different experimental setups. Finally, we propose that the ABCC1 transporter could be involved in export of S1P from human erythrocytes and that analysis of plasma S1P from patients treated with inhibitors of ABCC1 could be of clinical interest.

## Materials and Methods

### Measurement of S1P

Concentration of S1P in bovine serum albumin, HDL + apoM or HDL-apoM was performed as previously described^20^.

### S1P export from erythrocytes

The protocol was modified from Kobayashi *et al*.^17^. Fresh blood samples from healthy human volunteers were drawn in K_3_EDTA vials and centrifuged 10 min at 3,000 rpm at RT. Plasma and buffycoat was discarded and erythrocytes were pooled. Pooled erythrocytes (2.5 μl) were mixed with 1 ml Buffer A (20 mM HEPES, 3.3 mM NaH_2_PO_4_, 2.9 mM KCl, 1 mM MgCl_2_, 138 mM NaCl, 1 mg/ml glucose, pH 7.4) for 5 min at 37 °C and 500 rpm. ^3^H-sphingosine (20 pmol, specific activity 20 Ci/mmol) was added to the erythrocytes and incubated 5 min at 37 °C and 500 rpm. After incubation, the erythrocytes were centrifuged 5 seconds at 12,000 g and RT, and the pellet was washed twice with 200 μl Buffer A and resuspended in 10 μl Buffer A. The resuspended erythrocytes (2 μl) were added to tubes containing 200 μl acceptor diluted in Buffer A (total protein concentration 0.1–20 μg/ml) and incubated at 500 rpm and 37 °C for 40 min unless otherwise indicated. Total protein concentration of each acceptor was determined with Pierce BCA Protein Assay Kit (23225, Thermo Scientific). Finally, the erythrocytes were pelleted at 12,000 g for 5 seconds and the supernatants were transferred to new tubes for extraction. The pellets were washed twice with 200 μl Buffer A and resuspended in 25 μl ethanol for extraction.

### Extraction and quantification of ^3^H-sphingosine and ^3^H-S1P

Aliquots of the supernatants (150 μl) and the pellets were mixed with 150 μl chloroform:methanol (1:2 v/v) and sonicated in a water bath for 5 min followed by 2 min vortex. Then, 1 M NaCl (100 µl), 3 M NaOH (10 µl) and chloroform (100 µl) was added followed by 5 min vortex and 2 min centrifugation at 13,000 g and RT. This separates S1P into the upper aqueous phase and sphingosine into the lower organic phase. The aqueous phase was transferred to clean tubes and the organic phase was re-extracted with chloroform:methanol as above. The two aqueous phases combined (approximately 600 μl) and the organic phase (approximately 400 μl) were added to 10 ml OptiPhase (Perkin Elmer) and counted in a liquid scintillation counter. The amount of radioactivity in each sample was expressed as % of the total cpm in the aqueous and organic phases from supernatant and pellet combined.

The efficacy of the extraction protocol was tested by extraction of samples with fixed amounts of ^3^H-labelled sphingosine and S1P, respectively. The recovery of S1P in the aqueous phase was 99% and the recovery of sphingosine in the organic phase was 92%. Subsequently, to decrease the amount of radioactivity in the aqueous phase resulting from impurities, ^3^H-sphingosine was ‘purified’ by extraction and isolation of the organic phase, which was then evaporated and re-dissolved in ethanol. The ‘purified’ ^3^H-sphingosine was used in all experiments.

### Gel filtration

Supernatants from three individual S1P export assays (acceptor concentration 10 µg/ml of HDL-apoM and HDL + apoM or 500 µg/ml albumin) were pooled, mixed with 100 µl PBS + 0.1 g/l EDTA (700 µl total) and sterile filtered through a 0.45 µm filter. The pool (500 µl) was injected into a Superose 6 10/300 GL column (17-5172-01, Pharmacia Biotech) and run with PBS + 0.1 g/l EDTA at a flow of 0.3 ml/min. Fractions of six droplets were collected in a 96-well plate and selected fractions corresponding to a range of HDL particle sizes as well as unbound S1P were extracted with chloroform:methanol and quantified for ^3^H-S1P as described above. A second experiment was performed using 10 µg/ml HDL-apoM as acceptor as described above. After isolation of supernatants and prior to gelfiltration, the supernatants were incubated for 10 minutes at 37 °C in the presence of 500 µg/ml albumin.

To verify protein content, subsets of gel-filtration fractions (60 µl) were subject to trichloracidic precipitation prior to 12% SDS-PAGE and silver staining analysis.

### ABC-transporter inhibition assays

The S1P export assay was performed as described above with the addition of 5 min incubation at 37 °C with the inhibitors (concentration range 0.01–1 mM) prior to addition of acceptor. Probenecid and MK-571 was dissolved in buffer A, Vanadate was dissolved in H_2_O adjusted to pH 10 with NaOH, and Verapamil was dissolved in ethanol. The amount of solvent was below 1.5% of the total reaction volume.

### Production of recombinant apoM protein

Human apoM protein without the signal peptide (amino acids 1–21) and with an 8.5 kDa N-terminal Profinity eXact^TM^ tag was cloned and expressed in CHO cells by Evitria AG (Schlieren, Switzerland). Recombinant apoM protein was purified from cell supernatants by Profinity eXact^TM^ purification system (BioRad) according to the manufacturer’s protocol. The purified recombinant apoM was assessed by coomassie-stained SDS-PAGE, and western blot. Folding and biological activity of the recombinant protein was tested by the ability of S1P and palmitic acid to bind and quench the intrinsic tryptophan fluorescence of apoM as described^20,28^. S1P and palmitic acid was dissolved in chloroform/methanol/dimethyl-amine (40 wt. % in H_2_O) in a 5:15:3 ratio to a stock concentration of 2.6 mM. Purified recombinant apoM protein specifically binds S1P (see Supplemental Fig. S5).

### Purification of HDL with and without apoM

Mouse and human HDL (1.063 < *d* < 1.21) was isolated by sequential fixed density ultracentrifugation of plasma from ApoM-Tg^H^ ^24^, ApoM^−/−^ mice^24^, and healthy human donors. Ultracentrifugation was performed at 50,000 rpm and 4 °C for 16 hours (*d* = 1.063) or 24 hours (*d* = 1.21) in a Beckman Optima LE-80K ultracentrifuge with a Ti 50.4 rotor (Beckman Coulter). Density was adjusted with NaBr solutions containing 0.1 g/l EDTA and isolated lipoprotein fractions were dialyzed against PBS with 0.1 g/l EDTA. Total protein concentration in the isolated fractions was determined with Pierce BCA Protein Assay Kit (23225, Thermo Scientific).

Antibodies against human apoM were generated in mice by six sequential immunizations (interval of 2 weeks) with 100 µg recombinant apoM protein in incomplete Freund’s adjuvant. The mouse recipient obtaining highest antiserum reactivity (tested by indirect ELISA screening using purified HDL from apoM-Tg^H^ mice as antigen) was selected and boosted by a single injection before extraction and fusion of splenocytes. Indirect ELISA of cell supernatants with purified HDL from apoM-Tg^H^ mice as antigen identified 29 positive hybridoma clones. One specific antibody clone(19–01) was chosen and Protein-G purified antibodies from the cell culture supernatant were used for generation of an affinity column for human apoM. The antibody was immobilized by coupling to 1 ml HiTrap NHS-activated agarose resin (17-0716-01, GE Healthcare). Purified HDL (10 ml, ~42 mg total protein) from human plasma was passed over the antibody-coupled resin column. ApoM-containing HDL particles were subsequently eluted from the column by 0.1 M glycine, pH 2.2. The column was washed with 5 ml H_2_O followed by 5 ml tris-buffered saline. The procedure was repeated until apoM was undetectable in the flow-through (three times in total). Total protein concentration in the eluate and flow-through was determined by Pierce BCA Protein Assay Kit (23225, Thermo Scientific). The HDL + apoM and HDL-apoM particles were assessed with silver staining of SDS-gels, western blot against human apoM and ELISA measurement of human apoM^29^. The absence of S1P in HDL-apoM particles was confirmed with HPLC analysis^20^.

### Mice

Abcc1−/− (FVB.129P2-*Abcc1a* ^*tm1Bor*^ N12) mice and their wildtype controls (FVB.129) were purchased from Taconic and blood samples were collected from the tail vein. The mice were housed in a temperature-controlled facility with a 12-hour light/dark cycle at the Panum Institute, University of Copenhagen with water and chow diet *ad libitum*. All experiments were approved by The Animal Experiments Inspectorate, Ministry of Environment and Food of Denmark, and performed in accordance with appropriate guidelines and regulations from the The Animal Experiments Inspectorate.

### Ethics

All blood samples were collected from informed healthy volunteers. The samples were anonymized and materials were pooled from all individuals before further analysis. This was done in agreement with guidelines from the Regional Committees on Health Research Ethics for Region Hovedstaden, Denmark.

### Statistics

Statistical analyses were performed with GraphPad Prism version 4.03 (GraphPad Software, Inc.). Student’s t-test was used to compare data points obtained with different acceptor molecules at similar concentrations. P-values ≤ 0.05 were considered statistically significant. All experiments were conducted in duplicate or triplicate and data are presented as mean ± SEM.

## Electronic supplementary material

Supplementary information
