# Supplementary material for: Apolipoprotein M mediates sphingosine-1-phosphate efflux from erythrocytes
Source: Sci Rep. 2017 Nov 8;7:14983. doi: 10.1038/s41598-017-15043-y (PMC5678177; doi:10.1038/s41598-017-15043-y)
Supplement: Supplementary file 1 — Supplementary information [file 41598_2017_15043_MOESM1_ESM.pdf]

Apolipoprotein M mediates sphingosine-1-phosphate efflux from erythrocytes

Pernille M. Christensen[1,2], Markus H. Bosteen[1,2], Stefan Hajny[1,2], Lars B. Nielsen[1,3*], and Christina Christoffersen[1,2*]

[1] Department of Clinical Biochemistry, Rigshospitalet, Blegdamsvej 9, 2100 Copenhagen, Denmark.

[2] Department of Biomedical Sciences, University of Copenhagen, Blegdamsvej 3B, 2200 Copenhagen, Denmark. [3] Department of Clinical Medicine, University of Copenhagen, Blegdamsvej 3B, 2200 Copenhagen, Denmark.

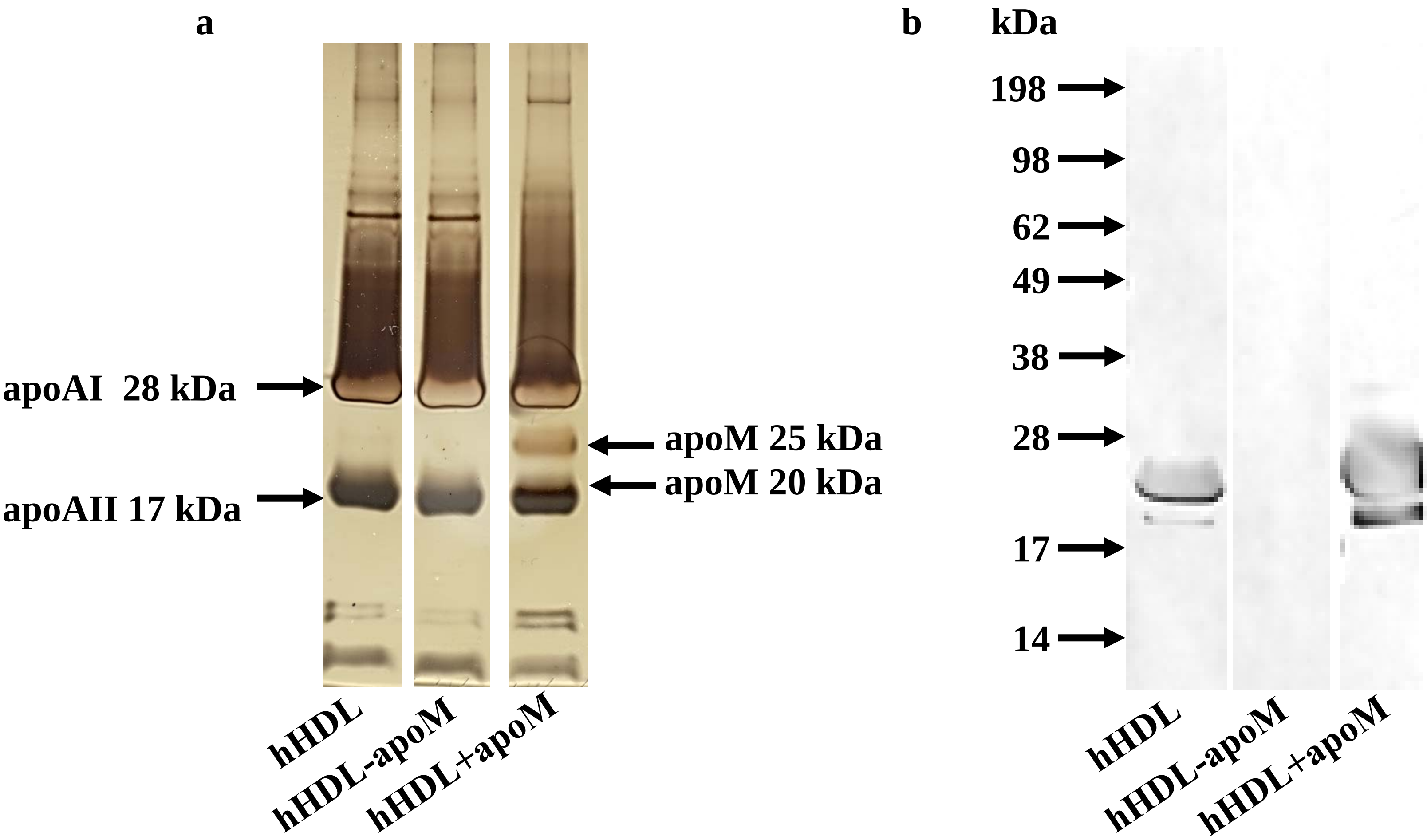

Figure S1. Characteristics of human HDL±apoM. Human HDL±apoM was purified using an immunoaffinity column. **(a)** Silverstained SDS gel loaded with 10 µg total protein of hHDL used for immunoaffinity-purification, purified hHDL-apoM and purified hHDL+apoM. The two different sizes of apoM correspond to glycosylated and non-glycosylated apoM. **(b)** Western blot for hapoM. For hHDL and hHDL-apoM 26 µg total protein was loaded and for hHDL+apoM 16 µg total protein was loaded.

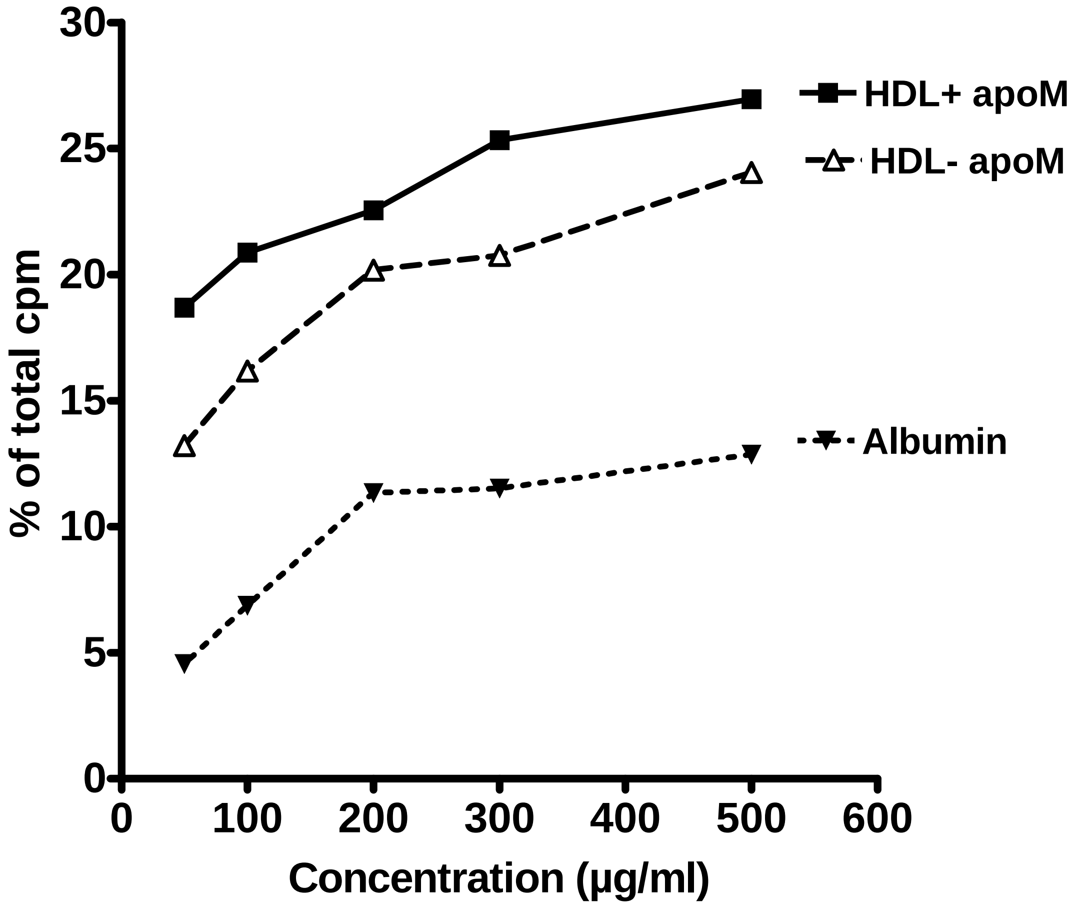

Figure S2. Incubation of erythrocytes with increased amount of protein. Export of S1P from erythrocytes incubated with HDL+apoM, HDL-apoM, or albumin. Total protein concentration varied from 50-500 µg/ml and samples were incubated 40 minutes. Results are expressed as % of the total cpm in supernatants and pellets combined.

Figure S2, Christensen et al

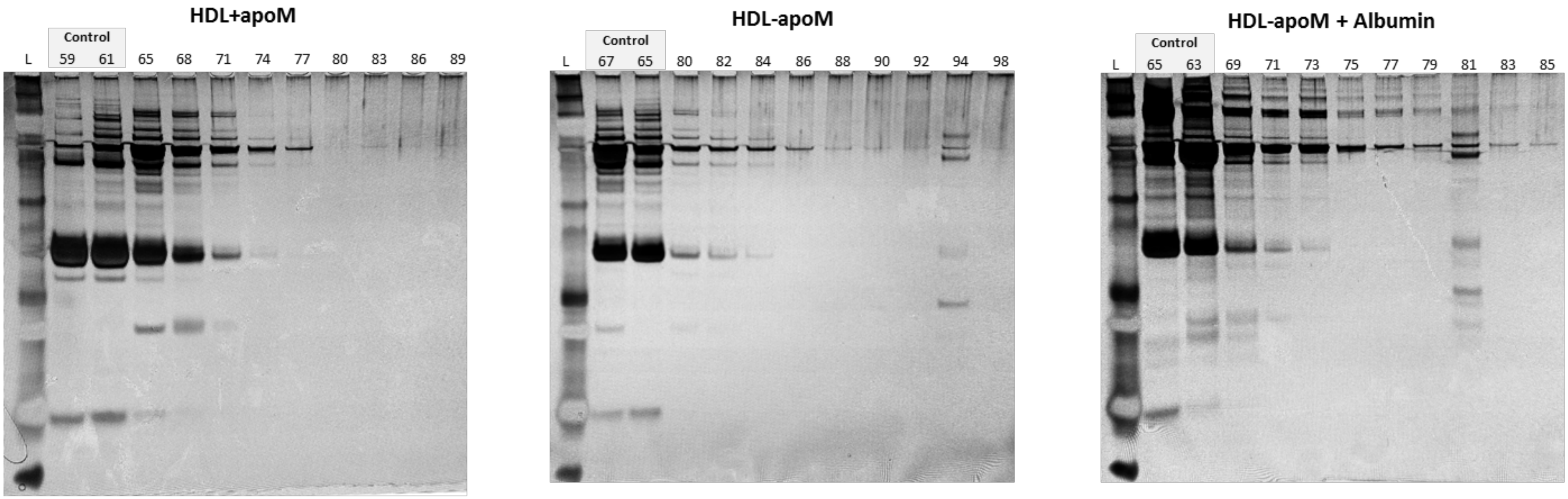

Figure S3. Protein content of HDL±apoM after erythrocyte assays. Supernatant were collected after S1P export assays using either HDL+apoM or HDL-apoM as acceptors. HDL-apoM + albumin represent an experiment were albumin was added the supernatant after export assays. The supernatants were subjected to FPLC analysis, and 96 fractions were collected. Gel filtration fractions (60 µl) were loaded on a 12% SDS gel after trichloracidic precipitation. The number indicated at the lane refer to the fractions number collected during FPLC. The proteins were visualized by silverstaining.

Figure S3, Christensen et al

a

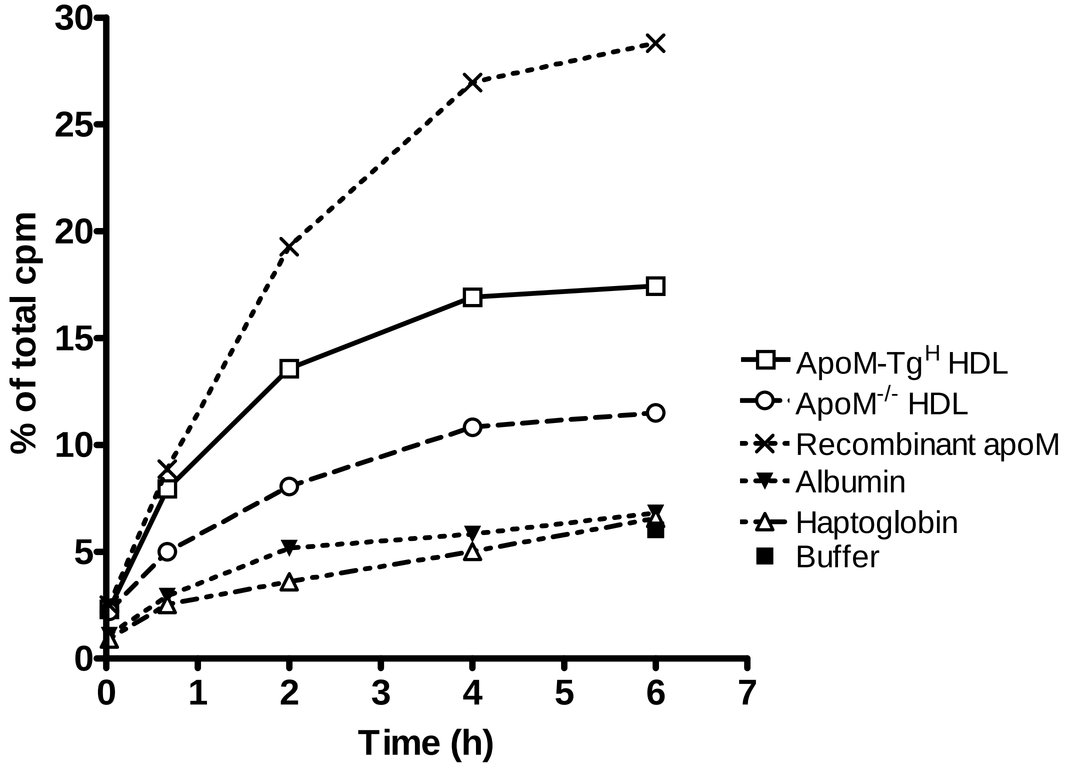

b

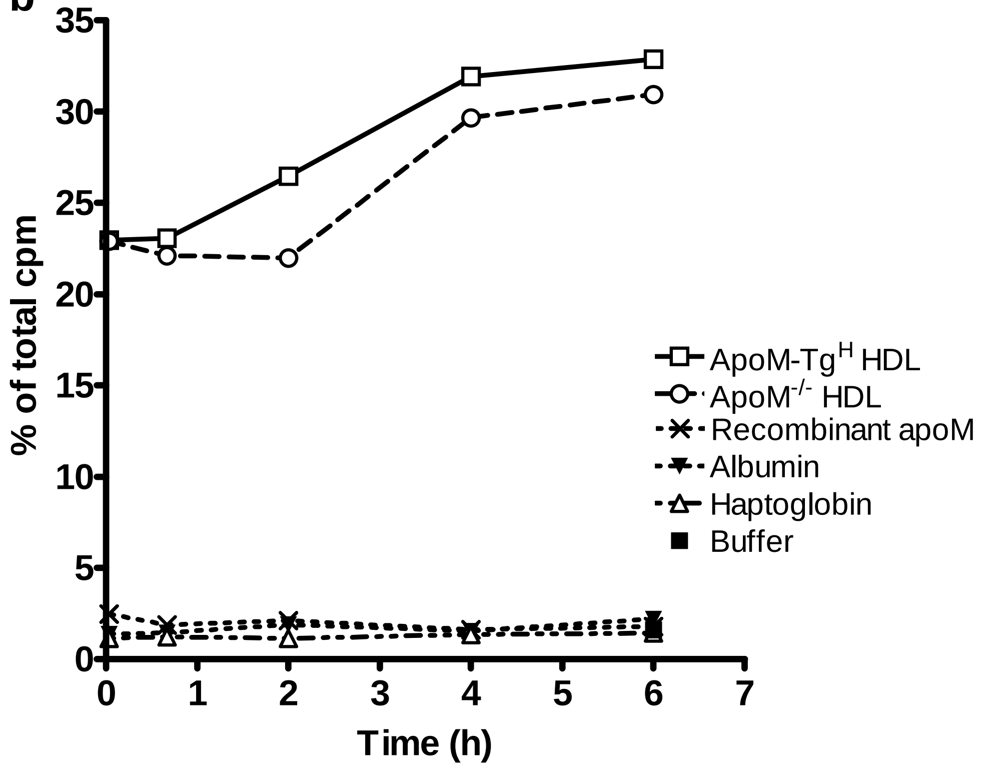

Figure S4. Long-term incubation of erythrocytes. Export of S1P **(a)** and sphingosine **(b)** from erythrocytes incubated with HDL from apoM transgenic mice (Tg^H), HDL from $apoM^{-/-}$ mice, recombinant apoM protein, albumin, haptoglobin, or assay buffer without any protein. Total protein concentration 20 µg/ml and samples were incubated 2 minutes to 6 hours. Results are expressed as % of the total cpm in supernatants and pellets combined.

Figure S4, Christensen et al

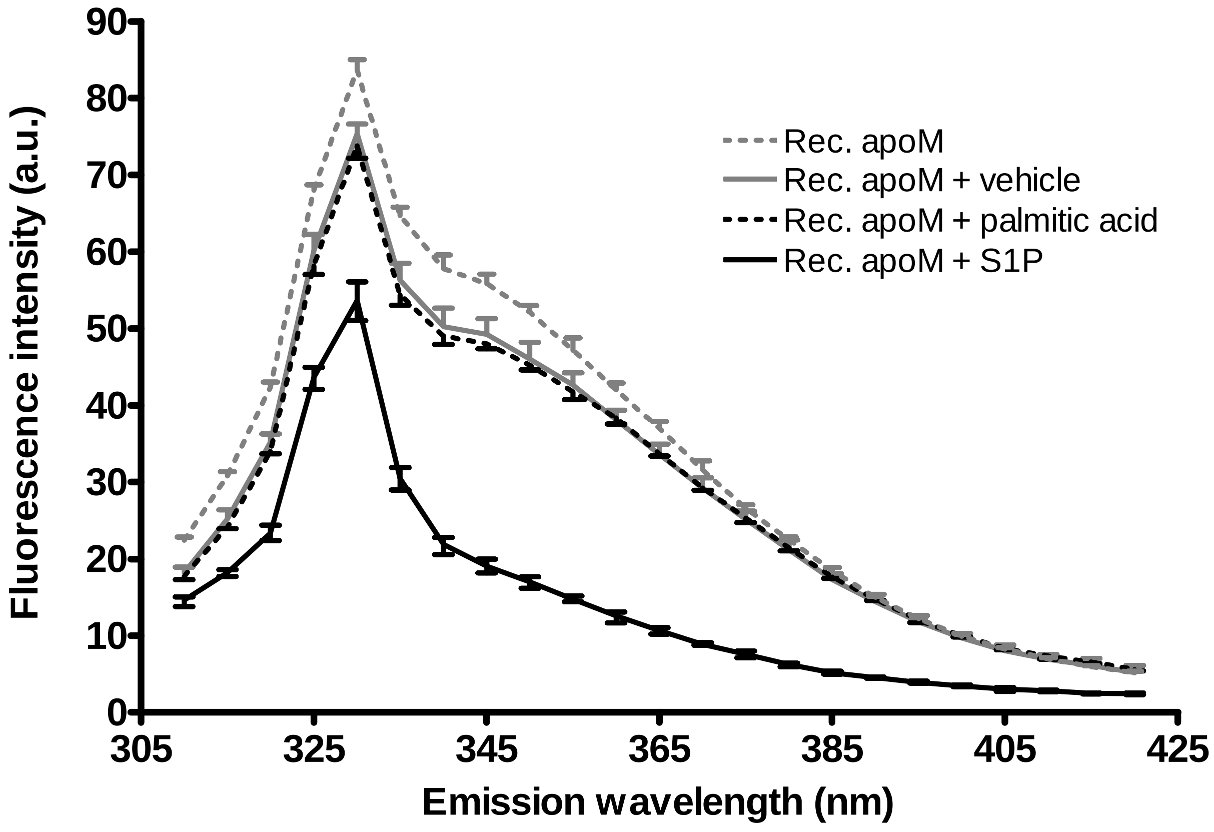

Figure S5. Recombinant apoM binds S1P. Binding of S1P to recombinant apoM was assessed by measurement of quenching of intrinsic fluorescence from apoM. Tryptophan fluorescence emission (recorded at 310-420 nm with 295 nm excitation) from recombinant human apoM (0.5 µmol/L) in H_2O (dashed grey line) and in the presence of vehicle control (solid grey line), 0.3 µM palmitic acid as negative control (dashed black line) or 0.3 µM S1P (solid black line). Lines represent means±range, n=4.

Figure S5, Christensen et al
